# Comparison of injecting drug users who obtain syringes from pharmacies and syringe exchange programs in Tallinn, Estonia

**DOI:** 10.1186/1477-7517-6-3

**Published:** 2009-02-20

**Authors:** Sigrid Vorobjov, Anneli Uusküla, Katri Abel-Ollo, Ave Talu, Kristi Rüütel, Don C Des Jarlais

**Affiliations:** 1 Estonian Drug Monitoring Centre, National Institute for Health Development, Hiiu 42, 11619 Tallinn, Estonia; 2 Estonian Centre of Excellence in Behavioural and Health Sciences, Tiigi 78, 50410 Tartu, Estonia; 3 Department of Public Health, University of Tartu, Ravila 19, 50411 Tartu, Estonia; 4 Department of Infectious Diseases and Drug Prevention, National Institute for Health Development, Hiiu 42, 11619 Tallinn, Estonia; 5 Chemical Dependency Institute, Beth Israel Medical Center, 160 Water St, New York, NY, 10038 USA

## Abstract

**Background:**

Both syringe exchange programs (SEPs) and pharmacy sales of syringes are available in Estonia, though the current high incidence and high prevalence of HIV among injection drug users (IDUs) in Tallinn, Estonia requires large-scale implementation of additional harm reduction programs as a matter of great urgency. The aims of this report were to compare risk behavior and HIV infection and to assess the prevention needs among IDUs who primarily use pharmacies as their source of sterile syringes with IDUs who primarily use SEPs in Tallinn.

**Methods:**

A cross-sectional study using respondent-driven sampling was used to recruit 350 IDUs for an interviewer-administered survey and HIV testing. IDUs were categorized into two groups based on their self-reported main source for syringes within the last six months. Odds ratios with 95% CI were used to compare characteristics and risk factors between the groups.

**Results:**

The main sources of sterile needles for injection drug users were SEP/SEP outreach (59%) and pharmacies (41%). There were no differences in age, age at injection drug use initiation, the main drug used or experiencing overdoses. Those IDUs using pharmacies as a main source of sterile needles had lower odds for being infected with either HIV (AOR 0.54 95% CI 0.33–0.87) or HCV (AOR 0.10 95% CI 0.02–0.50), had close to twice the odds of reporting more than one sexual partner within the previous 12 months (AOR 1.88 95% CI 1.17–3.04) and engaging in casual sexual relationships (AOR 2.09 95% CI 1.24–3.53) in the last six months.

**Conclusion:**

The data suggest that the pharmacy users were at a less "advanced" stage of their injection career and had lower HIV prevalence than SEP users. This suggests that pharmacies could be utilized as a site for providing additional HIV prevention messages, services for IDUs and in linking IDUs with existing harm reduction services.

## Background

More than twenty-five years into the pandemic, HIV infection continues to spread worldwide. The most recent epidemic has emerged in Eastern Europe. In 2001, the Russian Federation reported 60.24 new HIV cases per 100,000 population; Estonia reported 49.17 and Latvia 33.29 [1]. Although the number of new cases in 2006 had declined to 27 per 100,000 in Russia and 13 in Latvia, the incidence rate in Estonia is still notably high at 50 per 100,000 [1]. Injection drug use is the main cause of this prolonged HIV outbreak and according to the Joint United Nations Programme on HIV/AIDS the sharing of needles and syringes (referred to collectively, for the purposes of this study, as "syringes") are the key factors in transmitting around 80% of HIV infections in this region [2]. Studies in other locations have shown that HIV infection among injection drug users (IDUs) may lead to self-sustained heterosexual transmission of the virus [3].

Although syringe exchange programs (SEP) have been shown to be an effective response to injection-related HIV risks, there are too few SEPs, with limited hours of operation, to meet the needs of the growing IDU population [3-5]. Previous studies have shown that pharmacies can be an alternative source for sterile syringes and a venue for providing other services to IDUs [6-10]. Pharmacies are already involved in providing treatment for addiction, dispensing methadone, supervising methadone consumption and providing information on drug misuse and HIV prevention [6,7,9]. Pharmacies have also participated in syringe exchange and distributing or selling low price kits containing injecting equipment [6,8,10]. However, data on the use of pharmacies as a source of sterile syringes in Eastern European countries and Russia is lacking.

Syringe exchange began in Estonia in 1997 [11]. At the end of 2006 there were 26 SEPs, located in high drug use areas (in Tallinn and in the North-Eastern part of Estonia) [12]. The SEPs provide additional services besides syringe exchange (health education, social welfare advice, referral for blood-borne virus testing, medical and drug treatment). Over-the-counter sterile syringes are available in Estonian pharmacies without prescription.

In a location with high HIV incidence and prevalence among IDUs, limited resources, and evidence of high risk injection practices among IDUs, we need to understand the factors associated with getting injection equipment from different sources. Previous studies of HIV among IDUs in Estonia have examined prevalence and risk behavior among the samples either as a whole or by demographic subgroups [13-16]. Our aims were to examine the levels of risk behavior and the levels of HIV infection and to assess the prevention needs among IDUs who primarily use pharmacies as their source of sterile syringes and to compare them with IDUs who primarily use SEPs in Tallinn.

## Methods

Respondent-driven sampling (RDS) [17,18] was used to recruit 350 current IDUs for a cross-sectional risk behavior survey and biological sample collection for HIV testing. The study was conducted in spring 2007, in Tallinn. Inclusion criteria were being 18 years or older, Russian or Estonian language speakers, use of injection drugs in the previous two months and ability to provide informed consent. The inclusion criterion of drug use within two months was used with the aim of recruiting current IDUs.

Recruitment began with the non-random selection of five "seeds" representing diverse IDU types (by gender, ethnicity, main type of drug used, engaging in sex for money and HIV status). Eligible participants were provided with coupons for recruiting up to three of their peers. Coupons were uniquely coded to link participants to their survey responses and biological specimens and for monitoring who recruited whom. Participants who completed the study received a primary incentive (a food coupon worth 6.4 EUR) for participation in the study and a secondary incentive (food coupons worth 3.2 EUR for each eligible person they recruited to the study). The RDS technique uses participants' social networks to access individuals who may not appear in public venues and are not in contact with service providers. Data collected using RDS can be generalized to the sampled population when information about recruitment patterns (who recruited whom), network connections and social network sizes are gathered and incorporated into the analysis of estimates and confidence intervals [19,20].

We used an interviewer-administered questionnaire, in a face-to-face interview setting, based on the WHO Drug Injecting Study Phase II survey (version 2b (rev.2)) [21]. In order to assess local conditions and to subsequently adjust the instrument to best fit Tallinn's IDUs, we gathered information during a rapid assessment in October, 2006. Questions were selected that would elicit data on demographics, drug use history, HIV risk behavior, HIV testing, access and utilization of harm reduction services. Interviews were held in confidence, in a room of the SEP, between the IDU participant and the interviewer. Recruitment was conducted and the survey administrated by a team of trained fieldworkers. The study protocol included pre- and post-HIV test counseling for study participants.

Venous blood was collected from participants and tested with commercially available kits for HIV antibodies (using Abbott IMx HIV-1/HIV-2 III Plus from Abbott Laboratories, Abbott Park, Illinois, USA) and hepatitis C virus (HCV) antibodies (using ETI-AB-HCVK-3 from DiaSorin S.p.A, Via Crescentino, 13040 Saluggia, Italy). HIV test kits have proved to have high sensitivity and specificity (> 99%) [22,23]. The testing was conducted at the HIV/AIDS reference laboratory of the Tallinn Merimetsa Hospital.

IDUs were categorized into two groups, pharmacy or SEP (which included IDUs who got their syringes from SEP outreach workers), based on their self-reported main source for syringes within the previous six months. Risk behaviours and characteristics were compared between the two groups.

Descriptive statistics, including mean, standard deviation (SD) and range were used for continuous variables. For categorical variables, percentages and absolute (n) frequencies are presented. Student's t-test was used for continuous variables and chi-square test for categorical variables to explore differences. Odds ratios (OR) and 95% confidence intervals (95% CI) together with p-values were used to compare characteristics and risk factors between groups. Multivariate analysis based on conceptual hierarchical framework [24] was conducted to explore factors associated with using pharmacies as a main source of syringes. We also included factors that reached a statistically significant p-value (p < 0.05). Adjusted odds ratios (AOR) were calculated using gender, age, employment status, duration of injection career and frequency of injecting per day as control variables in a logistic regression model. Analyses were carried out using Stata 9 software [25].

The Ethics Review Board at the University of Tartu approved the study procedures.

## Results

Three hundred and fifty IDUs completed the questionnaire. A total of 99% answered that they had received new and unused syringes during the previous six months. The sources for new and unused syringes in that six month period were: pharmacies, 80%; SEPs, 72%; SEP outreach workers, 37%; friends, 23%; other drug users, 6%; drug workers and drug agencies, 4%; sexual partners, 2%; and street vendors, 1% (multiple responses allowed).

Of the 350 current IDUs recruited in Tallinn 328 (94%) were retained for the further analysis: 59% (n = 195) reported using SEP/SEP outreach and 41% (n = 133) reported using a pharmacy as the main source for sterile needles. We excluded 22 participants who reported alternative main source for sterile syringes from the two under consideration.

Twenty eight (21%) of the IDUs in the pharmacy group used also SEP outreach workers as an additional source for new syringes, and 68 (51%) used the SEP site as an additional source of syringes. However, 59 (45%) of the IDUs in the pharmacy group reported that they had never used SEPs. 132 (68%) of the IDUs in the SEP category had also used a pharmacy as an additional source of syringes.

Table 1 presents the univariate comparisons of IDUs in the pharmacy and SEP groups. The majority of the participants were male. The ages of the two groups were similar (mean 26.3 years, range 17 to 54 years, SD = 5.6 for pharmacy workers and mean 26.9 years, range 17 to 50 years, SD = 5.7 for SEP users). There were no differences in ethnicity, marital status or educational level. There were modest statistical differences between the factors of employment and health insurance. Pharmacy users were more likely to have regular or temporary employment than SEP users (61% versus 48%, p = 0.024), and more likely to have health insurance (50% vs. 38%, p = 0.040).

**Table 1:** Univariate comparisons between factors^1 ^and main sources for new and unused syringes

	Pharmacy		SEP^2^				
				
Variable	n	%	n	%	OR	95% CI	p-value
**Gender:**							
Male	118	88.72	160	82.05	1.72	0.90–3.30	0.102
Female	15	11.28	35	17.95	1.0		
**Age:**							
< 20	12	9.02	9	4.62	2.41	0.91–6.35	0.075
20–24	41	30.83	60	30.77	1.23	0.68–2.23	0.486
25–29	49	36.84	70	35.90	1.26	0.71–2.24	0.420
> 30	31	23.31	56	28.72	1.0		
**Ethnicity:**							
Russian	106	84.80	168	87.05	0.83	0.44–1.58	0.571
Estonian	19	15.20	25	12.95	1.0		
**Marital status:**							
Single	96	72.73	156	80.83	0.63	0.37–1.07	0.087
Married or cohabiting	36	27.27	37	19.17	1.0		
**Educational level:**							
< 9 years	72	54.55	100	51.28	0.72	0.17–2.97	0.650
10–12 years	56	42.42	91	46.67	0.62	0.15–2.56	0.504
> 12 years	4	3.03	4	2.05	1.0		
**Main source of income in last 6 months:**							
Other	52	39.10	101	51.79	0.60	0.38–0.93	0.024
Regular or temporary job	81	60.90	94	48.21	1.0		
**Having health insurance:**							
No	65	50.00	120	61.54	1.0		
Yes	65	50.00	75	38.46	1.60	1.02–2.51	0.040
**Duration of injection career:**							
0–2 years	22	16.54	11	5.70	3.74	1.65–8.51	0.002
3–5 years	30	22.56	30	15.54	1.87	0.99–3.54	0.054
6–9 years	42	31.58	79	40.93	0.99	0.58–1.71	0.986
> 10 years	39	29.32	73	37.82	1.0		
**Frequency of injecting:**							
Less than daily	50	37.59	47	24.10	1.0		
Daily	83	62.41	148	75.90	0.53	0.33–0.85	0.009
**Intensity of injecting per day:**							
One	33	24.81	22	11.34	1.0		
More than one	100	75.19	172	88.66	0.39	0.21–0.70	0.002
**Main drug injected during last 6 months:**							
Fentanyl	98	74.24	166	85.13	0.50	0.29–0.88	0.015
Amphetamine	71	53.38	98	50.26	1.13	0.72–1.76	0.578
**Ever overdosed:**							
No	52	39.10	61	31.28	1.0		
Yes	81	60.90	134	68.72	0.71	0.45–1.12	0.144
**Sharing syringes during last 6 months:**							
No	82	62.12	128	65.98	1.0		
Yes	50	37.88	66	34.02	1.18	0.75–1.87	0.475
**Sharing paraphernalia during last 6 months:**							
No	102	76.69	153	79.27	1.0		
Yes	31	23.31	40	20.73	1.16	0.68–1.98	0.579
**Ever shared needles with someone known to be HIV positive:**							
No	38	29.46	34	18.48	1.0		
Yes	91	70.54	150	81.52	0.54	0.32–0.92	0.024
**Sharing needles with sexual partner during last 6 months:**							
No	30	60.00	45	67.16	1.0		
Yes	20	40.00	22	32.84	1.36	0.63–2.92	0.425
**Number of sexual partners during last 12 months:**							
None or one	63	47.37	116	60.42	1.0		
More than one	70	52.63	76	39.58	1.70	1.08–2.65	0.020
**Number of casual partners during last 6 months:**							
None	71	53.38	133	68.21	1.0		
One or more	62	46.62	62	31.79	1.87	1.19–2.95	0.007
**Condom use with casual partner during last 6 months:**							
Never	12	19.35	11	17.19	1.0		
Occasionally/Always	50	80.65	53	82.81	0.86	0.35–2.14	0.753
**Condom use with primary partner during last 6 months:**							
Never	32	48.48	45	47.37	1.0		
Occasionally/Always	34	51.52	50	52.63	0.96	0.51–1.79	0.889
**Self-reported STI (syphilis, gonorrhea, chlamydia, genital herpes):**							
No	119	89.47	173	88.72	1.0		
Yes	14	10.53	22	11.28	0.93	0.46–1.88	0.830
**Disease serostatus:**							
HIV+	61	45.86	123	64.06	0.48	0.30–0.75	0.001
HCV+	117	87.97	190	98.96	0.08	0.02–0.34	0.001
**Ever had HIV test:**							
No	22	16.54	25	12.89	1.0		
Yes	111	83.46	169	87.11	0.75	0.40–1.39	0.356
**Ever received drug treatment:**							
No	82	61.65	115	58.97	1.0		
Yes	51	38.35	80	41.03	0.89	0.57–1.40	0.627
**Ever been in prison:**							
No	57	42.86	75	38.46	1.0		
Yes	76	57.14	120	61.54	0.83	0.53–1.30	0.426

^1^Reference group SEP users.

^2^Includes Syringe Exchange Program outreach workers as the source of new and unused syringes.

There were no differences in mean age at IDU initiation between the SEP (18.4 years, range 10 to 42 years, SD = 4.8) and pharmacy users (19.1 years, range 9 to 39 years, SD = 4.4). However there were significant differences in terms of the proportion of new injectors (IDUs with 0 to 2 years of injecting) and frequency of injecting daily between the two groups (Table 1). Pharmacy users were more likely to be new injectors (16% vs. 6%, p = 0.002) and less likely to inject daily (62% vs. 76%, p = 0.009). They also reported lower injecting frequency on the last day they injected, with 75% of pharmacy users and 89% of SEP users reporting more than one injection per day (p = 0.002). There were fewer fentanyl users among pharmacy users (74% vs 85%, p = 0.015), but no differences in terms of either injecting amphetamine or experiencing drug related overdoses. There was no difference in sharing practices between the groups, except that fewer pharmacy users reported sharing syringes with HIV positive individuals (71% vs. 82%, p = 0.024). However, pharmacy users reported riskier sexual behaviors – with a higher proportion reporting more than one sexual partner (53% vs. 40%, p = 0.020) within the preceding year and more reporting casual sexual partners (47% vs. 32%, p = 0.007) within previous six months.

There were important differences in HIV/HCV serostatus between the groups: fewer IDUs in the pharmacy group were HIV seropositive (46% vs. 64.0%, p = 0.001) or HCV seropositive (88% vs. 99%, p < 0.001). There were no differences in HIV testing prior to the study, having received drug abuse treatment or having been in prison.

We used the SEP users as a reference group to calculate the AORs and 95% CI for the pharmacy group for injection risk behavior, HIV and HCV serostatus, sexual behaviour and contacts with harm reduction and health services, see Table 2. Pharmacy users had close to twice the odds for having, in the previous year, either more than one sexual partner (AOR 1.88 95% CI 1.17–3.04) or engaging in casual sexual relationships (AOR 2.09 95% CI 1.24–3.53) in the last six months. The pharmacy group had lower odds for being infected with either HIV or HCV (AOR 0.54 95% CI 0.33–0.87 and 0.10 95% CI 0.02–0.50, accordingly).

**Table 2:** Multivariate factors^1 ^related to pharmacy as main source for syringes^2^

Variable	AOR	95% CI	p-value
**Main drug injected during last 6 months:**			
Fentanyl	0.62	0.34–1.13	0.120
Amphetamine	1.13	0.71–1.80	0.609
**Ever overdosed:**			
No	1.0		
Yes	0.88	0.53–1.45	0.606
**Sharing syringes during last 6 months:**			
No	1.0		
Yes	1.42	0.87–2.32	0.159
**Sharing paraphernalia during last 6 months:**			
No	1.0		
Yes	1.33	0.76–2.34	0.312
**Ever shared needles with someone known to be HIV positive:**			
No	1.0		
Yes	0.70	0.40–1.23	0.214
**Sharing needles with sexual partner during last 6 months:**			
No	1.0		
Yes	1.48	0.65–3.36	0.346
**Number of sexual partners during last 12 months:**			
None or one	1.0		
More than one	1.88	1.17–3.04	0.009
**Number of casual partners during last 6 months:**			
None	1.0		
One or more	2.09	1.24–3.53	0.006
**Condom use with casual partner during last 6 months:**			
Never	1.0		
Occasionally/Always	1.01	0.69–1.47	0.962
**Condom use with primary partner:**			
Never	1.0		
Occasionally/Always	0.75	0.38–1.48	0.406
**Self-reported STI (syphilis, gonorrhea, chlamydia, genital herpes):**			
No	1.0		
Yes	0.82	0.39–1.74	0.613
**Disease serostatus:**			
HIV+	0.54	0.33–0.87	0.012
HCV+	0.10	0.02–0.50	0.005
**Ever been tested for HIV:**			
No	1.0		
Yes	1.11	0.56–2.17	0.766
**Ever received drug treatment:**			
No	1.0		
Yes	1.16	0.71–1.89	0.548
**Ever been in prison:**			
No	1.0		
Yes	1.11	0.67–1.86	0.680

^1^Reference group SEP users.

^2 ^Adjusted for age, gender, employment status, duration of injection career and intensity of injecting per day.

The low numbers of participants reporting exclusive pharmacy or SEP/SEP outreach use prohibited a meaningful analysis. Nevertheless, while comparing only SEP/SEP outreach (n = 66) to only pharmacy (n = 68) users we found similar results to those obtained in the analysis presented above in terms of employment, health insurance statuses, but shorter injecting careers, higher numbers of sexual partners and less frequent injections and lower HIV status of exclusively pharmacy users in comparison to exclusively SEP/SEP outreach (data not shown).

## Discussion

We found that the great majority of IDUs in Tallinn are using either SEPs or pharmacies as their primary source for sterile syringes. Over half the study participants (59%) mentioned using SEP, whereas 41% reported pharmacy as their main source of sterile syringes. A similar study conduced in Tallinn two years earlier, found lower proportions using SEP, with 46% reporting SEPs including outreach workers and 50% of the participants reporting pharmacies as their main sources for sterile syringes [26].

HIV prevalence was high among both those who used SEPs and those who used pharmacies as their primary source of sterile syringes. In addition, both groups reported an unacceptably high rate of sharing syringes, especially sharing with people who are known to be HIV positive. Additional measures to reduce sharing, particularly between people who are HIV positive and HIV negative are urgently needed in Estonia.

There were significant differences between IDUs primarily using pharmacies and those using SEPs. Also the comparison of IDUs who used only SEPs or only pharmacies showed the same results suggesting that there are two distinct groups of IDUs with different risk profiles using pharmacies and SEPs. Pharmacy users were more likely to report multiple sexual partners and casual sexual partners. Condom use with casual and main partners was, however, equally low in both groups. This finding suggests that interventions which increase awareness of the risks associated with sexual transmission of HIV are also needed.

Our findings suggest that IDUs in the initial stages of their injection careers use pharmacies. According to the univariate analysis, pharmacy users included a higher proportion of new injectors (those reporting injecting two years or less) and reported lower injection frequencies. Previous studies from other locations have reported similar results: pharmacy users tend to be younger [27-29], with shorter injecting careers [29,30] and lower injecting frequencies [29,30]. New injectors create special problems for HIV prevention. Studies have found higher rates of injecting risk behavior and a higher incidence of blood-borne infections among new injectors [16,31-33]. Recent cohort studies confirm the same, recent initiates to injecting have a higher incidence of HIV and hepatitis C [34,35]. New injectors may increase the size of the local IDU population, increasing the need for prevention and treatment services. Also they may not self identify as IDUs, may not fully appreciate the need to protect themselves against HIV and other blood-borne diseases, and may find HIV prevention and drug services difficult to access.

In our study, nearly half the pharmacy users had never attended needle exchange, similar to a US study where pharmacy users were less likely to report recent SEP use [28]. This finding suggests that there could be IDUs who are beyond the reach of harm reduction services. One possible solution could be if pharmacies can take a role in linking IDUs with SEP services, especially for recent initiates. There are several examples in Europe, Australia, New Zealand, and the United States where pharmacies have been involved in providing services for drug users [8-10,27]. Despite some concerns for safety and about improperly discarded syringes, as well as undesired effects that drug users might have on the sensitivities of other business customers [8,28,36,37] it has been feasible to recruit pharmacists to provide services to IDUs [38,39] and to cultivate a public health perspective among pharmacists [36,40,41].

Our study has some limitations. The cross-sectional study design does not allow us to establish a causal relationship or a direction of causality. Second, we used a non-probability sample that may have implications for the representativeness of the study. However, we used RDS for recruitment in order to overcome some of the limitations of convenience sampling [17,18]. The statistical theory upon which RDS is based suggests that if peer recruitment proceeds through a sufficiently large number of waves, the composition of the sample will stabilize, becoming independent of the seeds from which recruitment began, and thereby overcoming any bias the nonrandom choice of seeds may have introduced [17,18]. Based on our results, the equilibrium state was achieved. Also, there might be residual (non-differential) misclassification due to the way we defined our study groups (pharmacy or SEP/SEP outreach users) leading to bias. Given that there were still statistically significant behavioral and HIV status differences between the groups, however, this supports rather than detracts from the contention that these two groups are significantly different.

Studies conducted in Russia and Eastern Europe have stressed the need for additional sources of syringes besides SEPs [42,43]. This study indicates the different profiles between IDUs who mainly use pharmacies for getting sterile syringes and those who mainly use SEPs. Data on the risk profiles of different groups of IDUs may be useful for developing targeted interventions. Encouraging pharmacies not only to sell sterile injection equipment to IDUs, as a regulated alternative to SEPs, but also to provide linkages to other services may be widely applicable in those areas where injecting drug use is a major driving force in HIV transmission.

## Conclusion

Our results show that pharmacy users were at a less "advanced" stage of their injection career than SEP users. This result reinforces the need for a comprehensive approach and the need for additional sources for acquiring syringes besides SEPs. Strategies to expand syringe access should be combined with other harm reduction services to make both sources more effective and easily utilized.

## Abbreviations

AOR: Adjusted Odds Ratio; HIV: Human Immunodeficiency Virus; HCV: Hepatitis C Virus; IDU: Injection Drug User; OR: Odds Ratio; SD: Standard Deviation; SEP: Syringe Exchange Program; WHO: World Health Organisation.

## Competing interests

The authors declare that they have no competing interests.

## Authors' contributions

AU, KAO, AT, KR designed the study. KAO, AT, KR supervised the data collection. SV, AU, KAO, DDJ planned the analysis of the manuscript. SV conducted the statistical analysis and wrote the first draft of the manuscript. All of the authors contributed to the final version of the manuscript.
